# Identifying serum microRNAs as biomarkers for endometriosis in adolescents and young adults

**DOI:** 10.1186/s12958-025-01502-z

**Published:** 2025-11-24

**Authors:** Alla Vash-Margita, Ramanaiah Mamillapalli, Karthik Pyneni, Davis Morgenstern, Hugh S. Taylor

**Affiliations:** Department of Obstetrics, Gynecology and Reproductive Sciences, Yale University School of Medicine, 310 Cedar Street, New Haven, CT 06520 USA

**Keywords:** Endometriosis, Adolescents, Young adults, MiRNA, Pelvic pain, Biomarker, MicroRNA

## Abstract

**Background:**

Endometriosis is a gynecological disorder that affects 190 million reproductive age women worldwide. Laparoscopic surgery is considered the gold standard to diagnose the disease, creating a barrier to diagnosis and leading to long delays in disease recognition. MicroRNAs may be useful in the diagnosis of this disease, however the ability to detect early disease in adolescents may be improved by identifying microRNAs specific to this population.

**Methods:**

This was a prospective clinical study evaluating adolescent patients with pelvic pain undergoing gynecologic surgery in an academic medical center. We enrolled 63 adolescent and young adult patients aged 13–26 years old undergoing gynecologic surgery between 2019 and 2024. Clinically relevant phenotypic and surgical information were recorded as well as evaluation of microRNAs abundance. We assessed microRNAs abundance by extracting total RNA from the serum samples and performed RNA-sequencing (RNAseq).

**Results:**

The mean age of adolescent women in the study was 16.3 and 15.9 for the endometriosis (*n* = 31) and control groups (*n* = 20), respectively. The mean BMI was 24.5 kg/m2 and 29.0 kg/m2 in the endometriosis and control groups, respectively. RNA-seq data analysis showed differential abundance of 859 microRNAs. Among 859 microRNAs, 488 were increased and 391 were decreased. We next selected those that were most distinct, with little overlap between subjects and controls. Four microRNAs were highly significantly increased while eighteen microRNAs were highly significantly decreased. We defined a signature of microRNAs that best distinguished subjects with endometriosis from controls.

**Conclusions:**

This is the first study to reveal the differential abundance of microRNAs specifically in adolescent patients with endometriosis. There are distinct differences from those identified in adult women with endometriosis. Our findings present a unique signature of microRNA found in the serum of adolescents with endometriosis. This finding may be useful as a noninvasive biomarker to diagnose early disease in adolescents. Non-invasive diagnosis may allow for early diagnosis and prevention of disease progression.

## Background

Endometriosis is a chronic inflammatory, estrogen-dependent condition defined as the presence of endometrial-like glands and stroma outside the uterine cavity. The pathophysiology of endometriosis is poorly understood with multiple factors proposed such as retrograde menstruation, hormonal, genetic, epigenetic, immunologic factors, stem cells as well as activation of the disease associated macrophages [1, 2]. Adolescent endometriosis is a highly prevalent condition, present in approximately 65% of adolescents undergoing laparoscopy for pelvic pain and in 75% of adolescents having laparoscopy for chronic pelvic pain that was unresponsive to treatment. One study describes that more than 50% of adults report that their symptoms started during adolescence with nearly a fifth of them reporting that symptoms began before age 20 and two thirds report onset before age 30 [3].

While diagnostic laparoscopy remains the gold standard for endometriosis diagnosis, a clinical diagnosis is often sufficient and, perhaps, even preferred in young patients [4]. Whether endometriosis is confirmed surgically or suspected clinically, the mainstay of treatment are the non-steroidal anti-inflammatory drugs and various hormonal and non-hormonal preparations that reduce estrogen production and prevent endometrial proliferation [5]. The optimal medical management of endometriosis in adolescents has not been identified; a recent study demonstrated that wide spectrum of regimens, including progestins, combined oral contraceptives, levonorgestrel intrauterine device, GnRH agonists and antagonists as well as combinations of these agents were a common practice [6]. Non-invasive biomarker of endometriosis may add clarity and shorten the time to diagnosis and treatment. Previously, we reported that six microRNAs miR-125b-5p, miR-150-5p, miR-342-3p, miR-451a, miR-3613-5p, Let-7b in women ages 18 years and older could be used as a predictive test for endometriosis [7, 8]. This is the first report showing that microRNA biomarkers can differentiate between endometriosis and other gynecological pathologies in a clinical setting [8].

There is considerable ongoing research attempting to identify a noninvasive diagnostic biomarker of endometriosis. A recent systematic literature review reports on 32 articles describing expression of 141 circulating microRNAs in patients with endometriosis, but studies are limited to participants 18 years of age and older [9]. There is concern about feasibility of the use of microRNAs as a biologic marker due to lack of replication and absence of validated differential expression. A review by Leonova et al. analyzed eighteen studies which collectively described 63 different microRNAs that differentially expressed in the circulation of women with endometriosis compared with controls, with only 14 microRNAs duplicated in one or more studies [10]. According to this review, downregulation of the circulating levels of miR-17, miR-20a, miR-139-3p, miR-141, miR-320a, miR-3613, and let- 7b was observed in more than one study, whereas, levels of miR-18a- 5p, miR-122, miR-125, miR-199a, miR-451a, miR-150, and miR342 were significantly upregulated in women with endometriosis compared with controls in more than one study as well [10–13]. Research dedicated to microRNAs as a potential biomarker specifically in adolescent population is lacking. Adolescent endometriosis is likely to be identified in the earlier stage of the disease and may be more readily detected with additional or different biomarkers. According to the Global Consortium of Investigators in Endometriosis, ongoing research in adolescent endometriosis should focus on biomarker specifically identified in adolescents [14].

The objective of this study was to evaluate whether microRNAs can be used as potential serum biomarkers for the diagnosis of adolescent endometriosis thus enabling early diagnosis. Here we report the differential expression of microRNAs in adolescent females with endometriosis.

## Methods

### Adolescent and young adult population

This study was approved by the Institutional review board of the Yale University School of Medicine (New Haven, CT, USA). The study was conducted at Yale-New Haven Hospital, a tertiary adult and children’s hospital. Investigators obtained written informed assent from patients undergoing surgery for suspected benign indications or chronic pelvic pain; consent was obtained from the guardian if patient was a minor and directly from the patient if 18 years of age or older. The study period was between March 2018 and December 2021. Adolescent and young adult (AYA) women aged 13–26 years were included. Exclusion criteria consisted of inability to sign consent in English, history of intellectual or physical disability thus being unable to provide assent/consent, refusal to participate and pregnancy. Enrollment was concurrent with surgical intervention in all but three participants. Surgery was performed due to known adnexal or other pathology or due to chronic pelvic pain. All patients who underwent surgery had laparoscopy as surgical approach. Total enrolment was 63 patients who were stratified to the endometriosis group (when surgical findings and/or histology confirmed endometriosis and also included three subjects who had endometriomas as determined by magnetic resonance imaging-MRI) and control group (no evidence of endometriosis but allowing other pathology, most commonly, paratubal cysts). Serum samples were collected from participants typically on the day of laparoscopy and microRNA expression analysis was performed blinded to the surgical findings. For participants who had intraoperative findings of endometriosis, the revised American Society of Reproductive Medicine (rASRM) classification was utilized to assigned stage of endometriosis as showed in Table 1. [15]. 

**Table 1:** Sociodemographic and medical characteristics of participants

Baseline characteristic	Endometriosis	Control	*P*-value
	(*N*=31)	(*N*=20)	
Age, years	16.3 ± 1.9	15.9 ± 3.2	0.152
BMI, kg/m^2^	24.5 ± 5.0	29.0 ± 8.9	0.016
Race			
White	26 (83.9)	15 (75.0)	
Black/African American	1 (3.2)	2 (10.0)	
Asian	2 (6.5)	0 (0)	
Other	2 (6.5)	3 (15.0)	
Phase of Menstrual Cycle			
Proliferative	8 (25.8)	6 (30.0)	
Secretory	3 (9.7)	5 (25.0)	
Unable to Determine***	20 (64.5)	9 (45.0)	
rASRM endometriosis stage			
1	18 (58.1)	-	
2	4 (12.9)	-	
3	4 (12.9)	-	
4	2 (6.5)	-	
No surgery	3 (9.7)	0 (0.0)	
Control Diagnosis			
No Abnormality	-	3 (15.0)	
Cystadenoma	-	3 (15.0)	
Teratoma	-	5 (25.0)	
Paratubal Cyst	-	7 (35.0)	
Mucinous Borderline Cystadenoma	-	1 (5.0)	
Cholecystitis	-	1 (5.0)	
Hormonal Treatment			
Combined OCP	13 (41.9)	6 (30.0)	
Progestin	11 (35.4)	3 (15.0)	
GnRH Agonist	1 (3.2)	0 (0)	
LNG IUD	1 (3.2)	1 (5.0)	
No treatment	3 (9.7)	10 (50.0)	
Transdermal patch	2 (6.5)	0 (0)	

*** Phase of the menstrual cycle could not be determined due to participants taking various hormonal preparations for menstrual suppression and contraception

Abbreviations: *BMI* Body Mass Index, *OCP* Oral Contraceptive Pill, *GnRH* Gonadotropin-Releasing Hormone, *rASRM* revised American Society for Reproductive Medicine, *LNG IUD* Levonorgestrel Intrauterine Device

### Sample collection

Samples were collected prior to surgery. Blood (5–10 ml) was drawn from the subjects and collected in sterile tubes (BD, Franklin Lakes, NJ, USA). Serum was collected immediately by centrifuging at 2500 rpm for min at 4 °C and stored at −80 °C.

### MicroRNA seq quality control

Total RNA was extracted from the serum samples using Quiagen kit miRNeasy Mini kit (cat. #17004, Qiagen, Germantown, MD, USA) and subjected to RNA. Seq. Analysis. Total RNA quality was determined by estimating the A260/A280 and A260/A230 ratios by nanodrop. RNA integrity is determined by running an Agilent Bioanalyzer gel or Agilent Fragment Analyzer, which measures the ratio of the ribosomal peaks. Samples with RIN values of 7 or greater are recommended for library prep.

### MicroRNA seq library Prep

Qiagen microRNA Library Prep Kit (Cat# 331505) was used for the miRNA Seq library preparation. RNA was normalized to a starting input between 1ng-500ng. Adapters were ligated sequentially to the 3’ and 5’ ends of microRNAs prior to cDNA synthesis with unique molecular identifiers (UMIs) assignment, cDNA cleanup, amplification, and final library cleanup. The library was run on the Agilent Tapestation and size selection was performed if there is excess adapter dimer. The library is then quantified by qRT-PCR using a commercially available kit (KAPA Biosystems).

### Flow cell preparation and sequencing

Sample concentrations were normalized to 1.2 nM and loaded onto an Illumina NovaSeq flow cell at a concentration that yields 10–25 million passing filter clusters per sample. Samples were sequenced using 100 bp paired-end sequencing on an Illumina NovaSeq according to Illumina protocols. The 8 bp unique dual index was read during additional sequencing reads that automatically follow the completion of read 1. Data generated during sequencing runs were simultaneously transferred to the Yale Center for Genome Analysis high-performance computing cluster. A positive control (prepared bacteriophage Phi X library) provided by Illumina was spiked into every lane at a concentration of 0.3% to monitor sequencing quality in real time. miRNA abundance was estimated using miRge3 using human small RNA reference database (https://sourceforge.net/projects/mirge3/files/miRge3_Lib/) [16]. The miRge3 analysis resulted in miRNA raw counts. The raw counts from mirGe3 analysis were used to estimate differentially expressed miRNAs.

### Primary analysis

Sample de-multiplexing and alignment to the human genome was performed using Illumina’s CASAVA 1.8.2 software suite. The data was checked for the sample error rate, confirming a rate of less than 2% and assuring that the distribution of reads per sample in a lane was within standard tolerance.

### Data analysis and storage

Differentially expressed miRNAs were identified using DESeq2 (v 1.22.1) [17]. Wald test was used for comparing the groups. Benjamini-Hochberg (BH) method was used to control the false discovery rate (FDR). We used p-value < 0.05 as a cutoff for significantly expressed miRNAs.

Signal intensities were converted to individual base calls during a run using the system’s Real Time Analysis (RTA) software. Base calls are transferred from the machine’s dedicated personal computer to the Yale High Performance Computing cluster via a 1 Gigabit network mount for downstream analysis.

## Results

During the study period we recruited 63 participants of which 12 were excluded due to low yield of microRNAs in the samples. We detected no miRNAs in these samples as miRNAs might have degraded during sample processing or was otherwise not detected. The final sample included 51 participants, 31 of which were designated to the endometriosis group and 20 assigned to the control group as showed in Table 1.. In the endometriosis group 28 (90%) patients had surgical intervention and all 20 patients in the control group had surgery. Patients in the endometriosis group who did not have surgery had evidence of endometriosis on pelvic MRI. All surgeries were done laparoscopically by surgeons highly skilled in recognition of adolescent endometriosis. The indication for surgical intervention in the endometriosis group was chronic pelvic pain, with a diagnosis of endometriosis allowing inclusion. The most common reason for surgery in the control group was adnexal pathology as well as one subject who underwent elective cholecystectomy. Paratubal cysts were the most common adnexal pathology in the control group with seven (36.4%) participants having paratubal cysts followed by five cases (26.3%) of benign teratomas. There was no significant age difference between endometriosis and control group (16.3+/−1.9 vs. 16.0+/−3.2,). The majority of participants in both groups were White, including 26 (83.9%) in endometriosis group and 15 (78.9%) in control group. In line with previous studies, we observed a significant difference in participants’ Body Mass Index (BMI) at 24.5 kg/m^2^ ± 5.0 for the endometriosis group and 28.4 kg/m^2^ ± 8.6 in control group (*p* = 0.028). The vast majority of participants in endometriosis group had stage I endometriosis based on the rASRM staging criteria [15]. We were unable to determine phase of the menstrual cycle in 20 out of 31 participants with endometriosis as they were treated with various hormonal preparations for menstrual suppression. Menstrual phase of the cycle was available in only 9 out of 20 controls due to majority of the participants using hormonal preparation for various reasons. The full characteristics of the adolescent subjects who participated (*N* = 63) in this study, including control subjects without endometriosis (*N* = 20) and subjects with endometriosis (*N* = 31), are shown in Table 1.

**Fig. 1:**
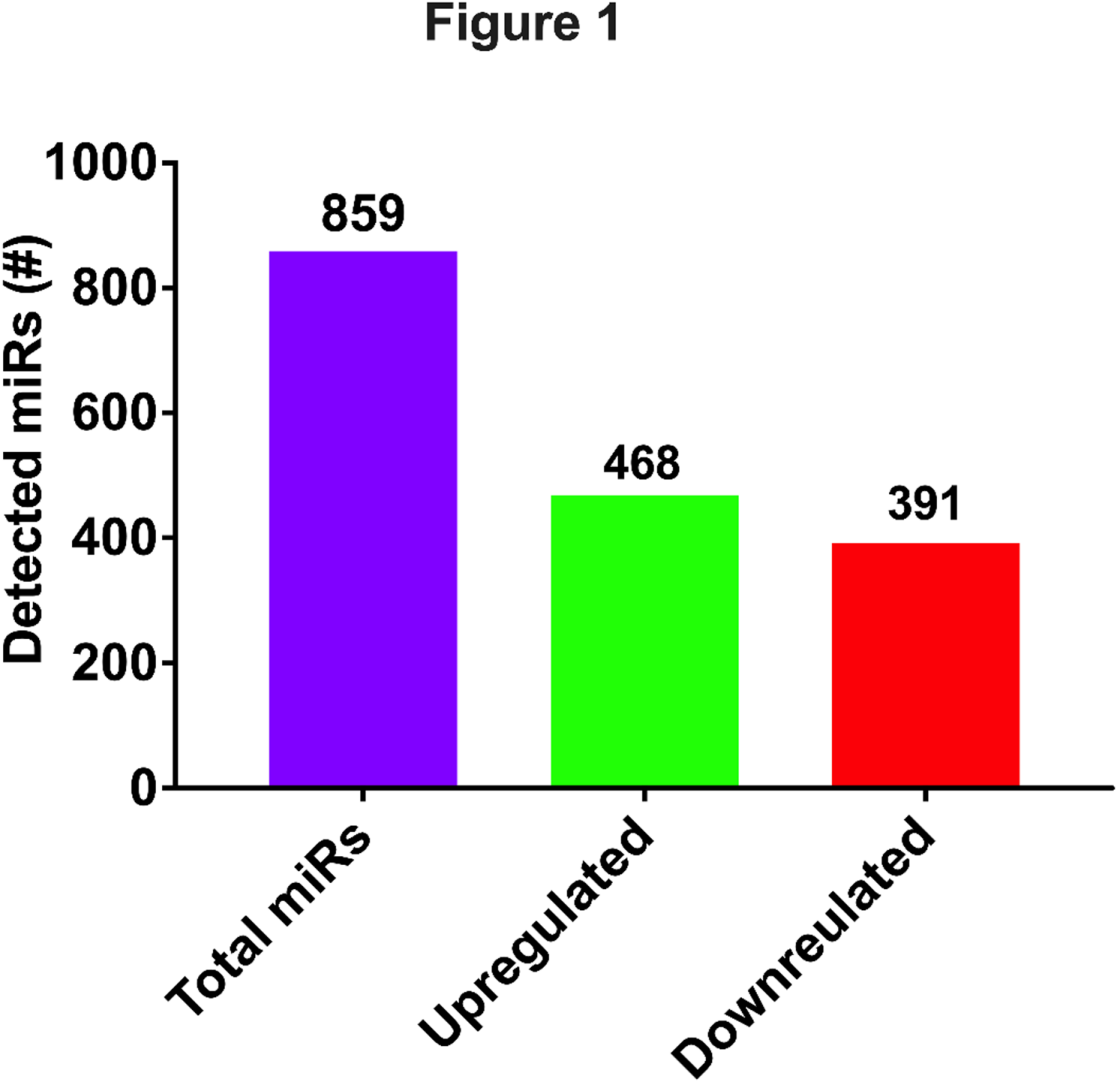
RNA-seq data identified a total 859 microRNAs detected in endometriosis. 468 microRNAs were upregulated and 391microRNAs were downregulated compared to controls without endometriosis

Analysis of microRNA-sequencing data demonstrated a total of 859 microRNAs in the serum of adolescents with endometriosis (Fig. 1).

In both the control group as well as the endometriosis group similar microRNAs were identified, however in the disease condition some of them were differentially abundant compared to the control group. Among 859 microRNAs detected, 468 microRNAs were increased while 391 were decreased compared to controls. Of these most showed small differences in abundance with considerable overlap between groups. This is consistent with our previously reported microarray analysis of microRNAs in the serum from adult women with endometriosis [7, 18]. We found that the majority of the microRNAs identified in adolescent patients with endometriosis were the same as previously identified in our prior microarray analysis of adults with endometriosis (Table 2).

**Table 2:** Common MicroRNAs between adult and adolescent endometriosis

No	microRNAs (matched)
1	Let-7b-5p
2	Let-7a-5p
3	Let-7c-5p
4	Let-7d-5p
5	Let-7e-5p
6	let-7f-5p
7	miR-135a
8	miR-125b-5p
9	miR-150-5p
10	miR-342-3p
11	miR-145-5p
12	miR-143-3p
13	miR-500a-3p
14	miR-451a
15	miR-18a-5p
16	miR-3613-5p
17	miR-6755-3p

We first examined microRNAs that were found in increased abundance among adolescents with endometriosis. Among upregulated microRNAs that we had not previously identified in adult endometriosis, there were four microRNAs that were significantly differentially expressed between control subjects and subjects with endometriosis, with sufficient separation to be considered as a potential biomarker in adolescents. These four microRNAs, specifically, miR-501-5p, miR-19b-3p, miR-3940-3p, and miR-1255b-5p, are listed in Table 3.

**Table 3:** Upregulated MicroRNAs

No.	microRNAs	Fold Change	*P*-Value
1	hsa-miR-501-5p*	1.8	0.034
2	hsa-miR-19b-3p*	1.6	0.023
3	hsa-miR-3940-3p*	1.5	0.037
4	hsa-miR-1255b-5p*	1.4	0.043

*Significant (*p* < 0.05)

Of these, miR-19b-3p has been reported in adult patients with EM-associated infertility, leaving three as potential adolescent specific biomarkers [19]. Among decreased microRNAs newly identified here, eighteen microRNAs were highly significantly differentially expressed and able to discriminate endometriosis from controls. These eighteen microRNAs are miR-2116-5p, miR-455-3p, miR-4686, miR-7850-5p, miR-34c-5p, miR-451b-5p, miR-1277-5p, miR-2355-3p, miR-5589-5p, miR-6513-5p, miR-3064-5p, miR-27b-5p, miR-9-5p, miR-760, miR-204-5p, miR-195-5p, miR-21-3p, and miR-34a-5p and are listed in Table 4.

**Table 4:** Downregulated MicroRNAs

No.	microRNAs	Fold Change	*P*-Value
1	hsa-miR-2116-5p*	-Inf	0.047
2	hsa-miR-455-3p*	-Inf	0.050
3	hsa-miR-4686*	-Inf	0.029
4	hsa-miR-7850-5p*	−32.4	0.036
5	hsa-miR-34c-5p*	−25.6	0.018
6	hsa-miR-451b-5p*	−17.6	0.011
7	hsa-miR-1277-5p*	−7.3	0.046
8	hsa-miR-2355-3p*	−6.3	0.037
9	hsa-miR-5589-5p*	−6.2	0.029
10	hsa-miR-6513-5p*	−5.9	0.005
11	hsa-miR-3064-5p*	−5.7	0.000
12	hsa-miR-27b-5p*	−4.1	0.009
13	hsa-miR-9-5p*	−3.1	0.044
14	hsa-miR-760*	−3.3	0.027
15	hsa-miR-204-5p*	−3.0	0.045
16	hsa-miR-195-5p*	−2.4	0.000
17	hsa-miR-21-3p*	−2.2	0.039
18	hsa-miR-34a-5p*	−2.0	0.039

*Significant (*p* < 0.05)

Among these 18 microRNAs, six microRNAs have been reported previously in adult women with endometriosis. MiR-34c-5p has a previously described role in endometrial receptivity [20] and a role in the development of endometriosis [21]. The other microRNAs miR-451 [21, 22], miR-204-5p [23], miR-21-3p [24], miR-34a-5p [25, 26], miR-19b-3p [19] also have a previously reported function in the pathophysiology of endometriosis. The remaining 11 microRNAs miR-2116-5p, miR-455-3p, miR-4686, miR-7850-5p, miR-1277-5p, miR-2355-3p, miR-5589-5p, miR-6513-5p, miR-3064-5p, miR-27b-5p, miR-9-5p, miR-760, miR-204-5p, miR-501-5p, miR-3940-3p, and miR-1255b-5p are uniquely differentially expressed in adolescent patients.

## Discussion

The diagnosis of endometriosis is typically delayed by 8–10 years, with the delay most pronounced in adolescents [27, 28]. The delay in diagnosis of endometriosis reaches nearly 14 years in women aged 9–19 years [29, 30]. The diagnostic process in adolescents is also more complex and the awareness of endometriosis in adolescents among medical professionals and caregivers of adolescents is low. Greene et al. described 4334 women with surgically confirmed endometriosis, reporting that women who first experienced symptoms as adolescents waited three times as long before seeking care compared to adults (6 vs. 2 years); it took longer before a diagnosis was made (5.4 vs. 1.9 years), and they reported not being taken seriously (65.2% vs. 48.9%) or told that nothing was wrong (69.6% vs. 49.8%) more often than women experiencing first symptoms as adults [31].

This is one of the few studies in young women with endometriosis to examine the differential abundance of circulating microRNAs in the blood. We identified a unique set of microRNAs in a subset of adolescent and young adult women with and without endometriosis.

RNA-sequence analysis of microRNAs in the serum of adolescent and young women with endometriosis demonstrated that a new set of microRNAs exist in circulation that are differed from microRNAs from the serum of adult women with endometriosis. Among four upregulated microRNAs that were significantly expressed in adolescent patients, three microRNAs miR-501-5p, maiR-3940-3p, and miR-1255b-5p were never reported previously in women with endometriosis. Among 18 microRNAs that were downregulated significantly in adolescent patients, six microRNAs miR-34c-5p [21, 32], miR-204-5p [23], miR-21-3p [24],, miR-451b-5p [33], miR-195-5p [34] and miR-34a-5p [25, 26] have been reported previously in women with endometriosis. In our study miR-9-5p expression was reduced significantly in contrast Zheng et al. [35] reported that it is upregulated in women with endometriosis. The remaining 11 microRNAs miR-2116-5p, miR-455-3p, miR-4686, miR-7850-5p, miR-1277-5p, miR-2355-3p, miR-5589-5p, miR-6513-5p, miR-3064-5p, miR-27b-5p, and miR-760 have never been reported previously in women with endometriosis. Therefore, this signature of microRNAs may have the potential to serve as a biomarker panel for adolescent patients with endometriosis. Expression of different microRNAs in adolescents might stem from earlier stages of the disease in younger patients. Our research complements discovery of 49 miRNAs that were differentially expressed between endometriosis cases and controls by Brady et al. [36]. In the same study authors completed neural network analysis that reveled 5 miRNAs in the final analysis (miR-542-3p, let-7b-3p, miR-548i, miR-769-5p, miR-30c-1-3p).

We reported previously the differential expression of circulating microRNAs in the serum of women with endometriosis [7, 8, 18]. Recently, we described a panel of six microRNAs as a biomarker to diagnose women with endometriosis [8]. Out of six microRNAs four microRNAs miR-125b, miR-150-5p, miR-342-3p, and miR-451a were significantly increased in patients with endometriosis while miR-3613-5p and let-7b were significantly decreased. Furthermore, our group demonstrated differential methylation of the Let-7b coding region in ectopic samples compared with eutopic endometrium from patients with endometriosis, suggesting the epigenetic nature of the differential regulation of microRNAs in endometriosis [37]. Circulating microRNA biomarkers, including miR-122 and miR-199a in serum [38] and miR-31 and miR-145 in plasma [39] have also reported as potential biomarkers to diagnose the adult women with endometriosis.

We have also demonstrated that targeting aberrantly produced microRNA is potential treatment of endometriosis [40, 41]. Targeting abnormally expressed microRNAs showed a robust respond to therapy with the reduction of lesion volume after endometriosis treatment [40, 42, 43]. MicroRNA analogs or inhibitors have potential as therapeutic agents in this disease, further demonstrating the utility of identifying the microRNA signature of adolescents with endometriosis. The distinct microRNAs signatures produced in adult women with endometriosis differ from microRNAs produced by adolescent women with endometriosis, suggesting the potential for therapy aimed specifically at this population.

Endometriotic lesions secrete some of these microRNAs directly, while some microRNAs are produced in response to the endometriosis by other tissues. Further, multiple organs in the body produce microRNAs in response to inflammation associated with endometriosis. Some of these microRNAs are released into the circulation where they have an effect on organs that may be far removed from the location of the endometriosis [1, 42, 44, 45]. Those microRNAs that are produced directly by endometriosis are more likely to be therapeutic targets. Other microRNA may account to the diffuse multiorgan effects of endometriosis and may be targeted to reduce these systemic effects.

Identifying microRNAs that are differentially expressed in adolescent endometriosis will broaden our understanding the role of microRNAs in the pathophysiology of early endometriosis. These microRNAs may provide novel biomarkers for endometriosis diagnosis and potential for treatment. While these findings must be confirmed in a large independent population, this work may produce a noninvasive serum biomarker of this chronic and debilitating disease at an early stage. Ability to diagnose endometriosis sooner will lead to improved quality of life and likely improved long-term outcomes as a result of more rapid and early treatment.

### Strengths and limitation of the study

This study has several strengths. Firstly, we evaluated participants prospectively and included other pelvic pathology, suggesting that these markers not only distinguish women with endometriosis from healthy individuals, but also can distinguish endometriosis from other common pathologies. Second, the investigators who were evaluating microRNA levels were blinded to the clinical or surgical diagnosis. Third, all but three subjects in the endometriosis group had surgically and histologically confirmed endometriosis and all participants in control group had surgery thus using the “gold standard” of surgical diagnosis.

We recognize a few limitations of our study. The limited number of participants precluded us from stratifying levels of microRNAs according to the severity or stage of the disease. The study was conducted at one institution comprising of participants of one geographic area. Our sample was uniform with predominance of Caucasian participants, which does not fully reflect the demographic makeup of the catchment area. Majority of participants used various hormonal preparations as the 1st line of treatment for pelvic pain with resultant amenorrhea which precluded us from accurately documenting the phase of menstrual cycle. Similarly, use of various hormonal preparations may have a confounding effect on the composition of circulating microRNAs. Lastly, our control group included patients with various histological adnexal masses which could have an impact on the composition of microRNA levels.

## Conclusions

This prospective cohort study carried out in adolescent women with endometriosis and identifies a distinct set of microRNAs that are differentially expressed compared to microRNAs reported in adult women with endometriosis. Further studies will include a larger sample with goal to validate these findings. Circulating microRNAs provides a hope for non-invasive method to diagnose endometriosis. Such a diagnostic tool would enable us to avoid surgery simply for diagnosis and to reduce the prolonged delay in detection of endometriosis. Earlier diagnosis could reduce the surgical risk, years of discomfort, hospitalizations and healthcare costs, disease progression and associated co-morbidities.

## Data Availability

No datasets were generated or analysed during the current study.

## Abbreviations

BMI: Body Mass Index
OCP: Oral Contraceptive Pill
GnRH: Gonadotropin-Releasing Hormone
rASRM: revised American Society for Reproductive Medicine
LNG IUD: Levonorgestrel Intrauterine Device

## References

[1] Taylor HS, Kotlyar AM, Flores VA. Endometriosis is a chronic systemic disease: clinical challenges and novel innovations. Lancet. 2021;397:839–52. 33640070 10.1016/S0140-6736(21)00389-5

[2] Lv H, Liu B, Dai Y, Li F, Bellone S, Zhou Y, et al. TET3-overexpressing macrophages promote endometriosis. J Clin Invest. 2024. 10.1172/JCI181839. 39141428 10.1172/JCI181839 PMC11527447

[3] Nnoaham KE, Hummelshoj L, Webster P, d’Hooghe T, de Cicco Nardone F, de Cicco Nardone C, Jenkinson C, Kennedy SH, Zondervan KT. Impact of endometriosis on quality of life and work productivity: a multicenter study across ten countries. Fertil Steril. 2011;96:366–e373368. 21718982 10.1016/j.fertnstert.2011.05.090 PMC3679489

[4] Agarwal SK, Chapron C, Giudice LC, Laufer MR, Leyland N, Missmer SA, Singh SS, Taylor HS. Clinical diagnosis of endometriosis: a call to action. Am J Obstet Gynecol. 2019;220:e354351–354312. 10.1016/j.ajog.2018.12.039 30625295

[5] ACOG Committee Opinion No. 760: Dysmenorrhea and Endometriosis in the Adolescent. Obstet Gynecol. 2018; 132:e249–e258. 10.1097/AOG.0000000000002978 30461694

[6] Li HJ, Esencan E, Song Y, Taylor HS, Cho Y, Vash-Margita A. Medical management of endometriosis in adolescent and young adult women: A review of 91 cases of Biopsy-Confirmed endometriosis. J Obstet Gynaecol Can. 2024;46:102562. 38759792 10.1016/j.jogc.2024.102562

[7] Cosar E, Mamillapalli R, Ersoy GS, Cho S, Seifer B, Taylor HS. Serum microRNAs as diagnostic markers of endometriosis: a comprehensive array-based analysis. Fertil Steril. 2016;106:402–9. 27179784 10.1016/j.fertnstert.2016.04.013

[8] Moustafa S, Burn M, Mamillapalli R, Nematian S, Flores V, Taylor HS. Accurate diagnosis of endometriosis using serum MicroRNAs. Am J Obstet Gynecol. 2020;223:e557551–557511. 10.1016/j.ajog.2020.02.050 32165186

[9] Vanhie A, Caron E, Vermeersch E, Tomassetti OD, Meuleman C, Mestdagh C. P, D’Hooghe TM: Circulating microRNAs as Non-Invasive Biomarkers in Endometriosis Diagnosis-A Systematic Review. Biomedicines 2024;12. 10.3390/biomedicines12040888 PMC11048084 38672242

[10] Leonova A, Turpin VE, Agarwal SK, Leonardi M, Foster WG. A critical appraisal of the circulating levels of differentially expressed microRNA in endometriosis†. Biol Reprod. 2021;105:1075–85. 34244742 10.1093/biolre/ioab134 PMC8599033

[11] Wang L, Huang W, Ren C, Zhao M, Jiang X, Fang X, et al. Analysis of serum microRNA profile by Solexa sequencing in women with endometriosis. Reprod Sci. 2016;23:1359–70. 27412772 10.1177/1933719116641761

[12] Papari E, Noruzinia M, Kashani L, Foster WG. Identification of candidate MicroRNA markers of endometriosis with the use of next-generation sequencing and quantitative real-time polymerase chain reaction. Fertil Steril. 2020;113:1232–41. 32482255 10.1016/j.fertnstert.2020.01.026

[13] Jia SZ, Yang Y, Lang J, Sun P, Leng J. Plasma miR-17-5p, miR-20a and miR-22 are down-regulated in women with endometriosis. Hum Reprod. 2013;28:322–30. 23203215 10.1093/humrep/des413 PMC3733164

[14] Rogers PA, Adamson GD, Al-Jefout M, Becker CM, D’Hooghe TM, Dunselman GA, Fazleabas A, Giudice LC, Horne AW, Hull ML, et al. Research priorities for endometriosis. Reprod Sci. 2017;24:202–26. 27368878 10.1177/1933719116654991 PMC5933154

[15] Revised American Society for Reproductive Medicine classification of endometriosis. 1996. Fertil Steril. 1997; 67:817–821. 10.1016/s0015-0282(97)81391-x 9130884

[16] Friedländer MR, Mackowiak SD, Li N, Chen W, Rajewsky N. miRDeep2 accurately identifies known and hundreds of novel MicroRNA genes in seven animal clades. Nucleic Acids Res. 2012;40:37–52. 21911355 10.1093/nar/gkr688 PMC3245920

[17] Love MI, Huber W, Anders S. Moderated estimation of fold change and dispersion for RNA-seq data with DESeq2. Genome Biol. 2014;15:550. 25516281 10.1186/s13059-014-0550-8 PMC4302049

[18] Cho S, Mutlu L, Grechukhina O, Taylor HS. Circulating MicroRNAs as potential biomarkers for endometriosis. Fertil Steril. 2015;103:1252–e12601251. 25772772 10.1016/j.fertnstert.2015.02.013 PMC4417410

[19] Li Y, Ye Y, Zhang H, Yang Y, Zhang N, Gao H, Wu R. MiR-19b-3p inhibits cell viability and proliferation and promotes apoptosis by targeting IGF1 in KGN cells. Gynecol Endocrinol. 2024;40:2425318. 39505692 10.1080/09513590.2024.2425318

[20] Cai H, Zhu XX, Li ZF, Zhu YP, Lang JH. MicroRNA dysregulation and steroid hormone receptor expression in uterine tissues of rats with endometriosis during the implantation window. Chin Med J (Engl). 2018;131:2193–204. 30203794 10.4103/0366-6999.240808 PMC6144856

[21] Yue Y, Lu B, Ni G. Circ_0001495 influences the development of endometriosis through the miRNA-34c-5p/E2F3 axis. Reprod Biol. 2024;24:100876. 38458026 10.1016/j.repbio.2024.100876

[22] Hawkins SM, Creighton CJ, Han DY, Zariff A, Anderson ML, Gunaratne PH, et al. Functional microRNA involved in endometriosis. Mol Endocrinol. 2011;25:821–32. 21436257 10.1210/me.2010-0371 PMC3082329

[23] Yi M, Wang S, Zhang X, Jiang L, Xia X, Zhang T, Fang X. Linc-ROR promotes EMT by targeting miR-204-5p/SMAD4 in endometriosis. Reprod Sci. 2023;30:2665–79. 36917423 10.1007/s43032-023-01204-0

[24] Zou W, Wang X, Xia X, Zhang T, Nie M, Xiong J, Fang X. Resveratrol protected against the development of endometriosis by promoting ferroptosis through miR-21-3p/p53/SLC7A11 signaling pathway. Biochem Biophys Res Commun. 2024;692:149338. 38043156 10.1016/j.bbrc.2023.149338

[25] Misir S, Hepokur C, Oksasoglu B, Yildiz C, Yanik A, Aliyazicioglu Y. Circulating serum miR-200c and miR-34a-5p as diagnostic biomarkers for endometriosis. J Gynecol Obstet Hum Reprod. 2021;50:102092. 33601073 10.1016/j.jogoh.2021.102092

[26] Ma Y, Huang YX, Chen YY. miRNA–34a–5p downregulation of VEGFA in endometrial stem cells contributes to the pathogenesis of endometriosis. Mol Med Rep. 2017;16:8259–64. 28990049 10.3892/mmr.2017.7677

[27] Janssen EB, Rijkers AC, Hoppenbrouwers K, Meuleman C, D’Hooghe TM. Prevalence of endometriosis diagnosed by laparoscopy in adolescents with dysmenorrhea or chronic pelvic pain: a systematic review. Hum Reprod Update. 2013;19:570–82. 23727940 10.1093/humupd/dmt016

[28] Hirsch M, Dhillon-Smith R, Cutner AS, Yap M, Creighton SM. The prevalence of endometriosis in adolescents with pelvic pain: a systematic review. J Pediatr Adolesc Gynecol. 2020;33:623–30. 32736134 10.1016/j.jpag.2020.07.011

[29] Pino I, Belloni GM, Barbera V, Solima E, Radice D, Angioni S, Arena S, Bergamini V, Candiani M, Maiorana A, et al. Better late than never but never late is better, especially in young women. A multicenter Italian study on diagnostic delay for symptomatic endometriosis. Eur J Contracept Reprod Health Care. 2023;28:10–6. 36287190 10.1080/13625187.2022.2128644

[30] Geysenbergh B, Dancet EAF, D’Hooghe T. Detecting endometriosis in adolescents: why not start from self-report screening questionnaires for adult women? Gynecol Obstet Invest. 2017;82:322–8. 27816976 10.1159/000452098

[31] Greene R, Stratton P, Cleary SD, Ballweg ML, Sinaii N. Diagnostic experience among 4,334 women reporting surgically diagnosed endometriosis. Fertil Steril. 2009;91:32–9. 18367178 10.1016/j.fertnstert.2007.11.020

[32] Luo Y, Wang D, Chen S, Yang Q. The role of miR-34c-5p/Notch in epithelial-mesenchymal transition (EMT) in endometriosis. Cell Signal. 2020;72:109666. 32353411 10.1016/j.cellsig.2020.109666

[33] Gao S, Liu S, Gao ZM, Deng P, Wang DB. Reduced microRNA-451 expression in eutopic endometrium contributes to the pathogenesis of endometriosis. World J Clin Cases. 2019;7:2155–64. 31531311 10.12998/wjcc.v7.i16.2155 PMC6718782

[34] Braza-Boïls A, Salloum-Asfar S, Marí-Alexandre J, Arroyo AB, González-Conejero R, Barceló-Molina M, García-Oms J, Vicente V, Estellés A, Gilabert-Estellés J, Martínez C. Peritoneal fluid modifies the MicroRNA expression profile in endometrial and endometriotic cells from women with endometriosis. Hum Reprod. 2015;30:2292–302. 26307093 10.1093/humrep/dev204

[35] Zheng J, Shao S, Dai C, Guan S, Chen H. miR-9-5p promotes the invasion and migration of endometrial stromal cells in endometriosis patients through the SIRT1/NF-κB pathway. Int J Clin Exp Pathol. 2020;13:1859–66. 32782715 PMC7414472

[36] Brady P, Yousif A, Sasamoto N, Vitonis AF, Fendler W, Stawiski K, Hornstein MD, Terry KL, Elias KM, Missmer SA, Shafrir AL. Plasma MicroRNA expression in adolescents and young adults with endometriosis: the importance of hormone use. Front Reproductive Health 2024, Volume 6–2024. 10.3389/frph.2024.1360417 PMC11043576 38665804

[37] Meixell DA, Mamillapalli R, Taylor HS. Methylation of microribonucleic acid let-7b regulatory regions in endometriosis. F S Sci. 2022;3:197–203. 35560017 10.1016/j.xfss.2022.03.001

[38] Maged AM, Deeb WS, El Amir A, Zaki SS, El Sawah H, Al Mohamady M, Metwally AA, Katta MA. Diagnostic accuracy of serum miR-122 and miR-199a in women with endometriosis. Int J Gynaecol Obstet. 2018;141:14–9. 29149541 10.1002/ijgo.12392

[39] Bashti O, Noruzinia M, Garshasbi M, Abtahi M. MiR-31 and miR-145 as potential non-invasive regulatory biomarkers in patients with endometriosis. Cell J. 2018;20:84–9. 29308623 10.22074/cellj.2018.4915 PMC5759684

[40] Sahin C, Mamillapalli R, Yi KW, Taylor HS. MicroRNA Let-7b: a novel treatment for endometriosis. J Cell Mol Med. 2018;22:5346–53. 30063121 10.1111/jcmm.13807 PMC6201226

[41] Li M, Zhou Y, Taylor HS. MiR-451a inhibition reduces established endometriosis lesions in mice. Reprod Sci. 2019;26:1506–11. 31354069 10.1177/1933719119862050

[42] Hu Z, Mamillapalli R, Taylor HS. Increased circulating miR-370-3p regulates steroidogenic factor 1 in endometriosis. Am J Physiol Endocrinol Metab. 2019;316:E373-82. 30576245 10.1152/ajpendo.00244.2018 PMC6459299

[43] Cosar E, Mamillapalli R, Moridi I, Duleba A, Taylor HS. Serum MicroRNA biomarkers regulated by Simvastatin in a primate model of endometriosis. Reprod Sci. 2018; 1933719118765971. 10.1177/1933719118765971 PMC6949973 29587611

[44] Zolbin MM, Mamillapalli R, Nematian SE, Goetz TG, Taylor HS. Adipocyte alterations in endometriosis: reduced numbers of stem cells and MicroRNA induced alterations in adipocyte metabolic gene expression. Reprod Biol Endocrinol. 2019;17:36. 30982470 10.1186/s12958-019-0480-0 PMC6463663

[45] Nematian SE, Mamillapalli R, Kadakia TS, Majidi Zolbin M, Moustafa S, Taylor HS. Systemic inflammation induced by micrornas: endometriosis-Derived alterations in Circulating MicroRNA 125b-5p and Let-7b-5p regulate macrophage cytokine production. J Clin Endocrinol Metab. 2018;103:64–74. 29040578 10.1210/jc.2017-01199

